# Electrostatics of non-neutral biological microdomains

**DOI:** 10.1038/s41598-017-11590-6

**Published:** 2017-09-12

**Authors:** J. Cartailler, Z. Schuss, D. Holcman

**Affiliations:** 1 Ecole Normale Supérieure, Applied Math and Computational Biology, IBENS 46 rue d’Ulm, 75005 Paris, France; 2 Department of Mathematics, Tel-Aviv University, Tel-Aviv, 69978 Israel; 3 Mathematical Institute, University of Oxford, Oxford, OX2 6GG United Kingdom

## Abstract

Voltage and charge distributions in cellular microdomains regulate communications, excitability, and signal transduction. We report here new electrical laws in a biological cell, which follow from a nonlinear electro-diffusion model. These newly discovered laws derive from the geometrical cell-membrane properties, such as membrane curvature, volume, and surface area. The electro-diffusion laws can now be used to predict and interpret voltage distribution in cellular microdomains such as synapses, dendritic spine, cilia and more.

## Introduction

Electro-diffusion in cellular microdmains remains difficult to study due to the absence of specific sensors and the theoretical hurdle of understanding the dynamics of charged particle in shaped domains. The diffusion of charged particles is largely influenced by the interaction of diffusing ions with the electrical field generated by all charges in the solution and possibly with external field.

The dielectric membrane of a charged biological cell also affects the electric field, because it creates image charges (see ref. 1 for an infinite plan). So far, only a few electro-diffusion systems are well understood. For example, although the electrical battery was invented more than 200 years ago, designing optimal configurations is still a challenge. On the other extreme, ionic flux and gating of voltage-channels^2^ is now well explained by the modern Poisson-Nernst-Planck (PNP) theory of electro-diffusion, because at the nanometer scale, cylindrical symmetry of a channel model reduces computations to a one-dimensional model for the electric field and charge densities in the channel pore^3–5^. However, cellular microdomains involve two- and three-dimensional neuronal geometry^7–11^, which makes the analysis of the PNP equations much more complicated than in the cylindrical geometry of a channel pore^12^.

We report here results about the distribution of charges and field, obtained from the analysis of the nonlinear PNP model of electro-diffusion in various geometries of microdomains in the absence of electro-neutrality. Although at this stage, we are still lacking experimental proofs of the nonelectro-neutrality at a scale of hundreds of nanometers, there is a clear unbalance in the concentrations of positive versus negative ions (see discussion below). This hypothesis should be considered as a prediction and is the basis of the present computations. In our model the entire boundary is impermeable to particles (ions) and the electric field satisfies the compatibility condition of Poisson’s equation. Phenomenological descriptions of the electro-diffusion, such as cable equations or the reduced electrical-engineering approximation by resistance, capacitance, and even electronic devices, are insufficient for the description of non-cylindrical geometry^13^, because they assume simple one-dimensional or reduced geometry.

## Results

### Electrostatic theory with no electro-neutrality

In the absence of electro-neutrality and with N charges distributed in a bounded domain Ω surrounded by a dielectric membrane, the PNP model for total charge *Q* = *zeN*, where *e* is the electron charge (1.6 · 10^−19^ C), *z* - the valence, and the charge density *ρ*(*x*, *t*) is given by ref. 14 for *x* ∈ Ω,

1 $$D[{\rm{\Delta }}\rho (x,t)+\frac{ze}{kT}\nabla (\rho (x,t)\nabla V(x,t))]=\,\frac{\partial \rho (x,t)}{\partial t}$$

2 $$D[\frac{\partial \rho (x,t)}{\partial n}+\frac{ze}{kT}\rho (x,t)\frac{\partial V(x,t)}{\partial n}]=0,x\in \partial {\rm{\Omega }}$$

3 $$\rho (x,\mathrm{0)}=q(x),x\in {\rm{\Omega }},$$

where *D* is the diffusion coefficient, *kT* represents the thermal energy and *V*(*x*, *t*) is the electric potential in Ω. It is the solution of the Poisson equation

4 $${\rm{\Delta }}V(x,t)=-\frac{ze\rho (x,t)}{\varepsilon {\varepsilon }_{0}}\,{\rm{for}}\,x\in {\rm{\Omega }}$$

with the boundary condition

5 $$\frac{\partial V(x,t)}{\partial n}=-\sigma (x,t)\,{\rm{for}}\,x\in \partial {\rm{\Omega }},$$

where the surface charge density *σ*(*x*, *t*) is defined on the boundary ∂Ω using the the electrical permitivity *εε*_0_ of the electrolyte solution. In the steady state,

6 $$\sigma (x)=\frac{Q}{\varepsilon {\varepsilon }_{0}|\partial {\rm{\Omega }}|}\mathrm{.}$$

Then (1) gives the density

7 $$\rho (x)=N\frac{{\rm{e}}{\rm{x}}{\rm{p}}\{-\frac{zeV(x)}{kT}\}}{{\int }_{{\rm{\Omega }}}{\rm{e}}{\rm{x}}{\rm{p}}\{-\frac{zeV(x)}{kT}\}dx},$$

hence (4) becomes

8 $${\rm{\Delta }}V(x)=-\frac{zeN\,{\rm{e}}{\rm{x}}{\rm{p}}\{-\frac{zeV(x)}{kT}\}}{\varepsilon {\varepsilon }_{0}{\int }_{{\rm{\Omega }}}{\rm{e}}{\rm{x}}{\rm{p}}\{-\frac{zeV(x)}{kT}\}dx},$$

and (5) gives the boundary condition

9 $$\frac{\partial V(x)}{\partial n}=-\frac{zeN}{\varepsilon {\varepsilon }_{0}|\partial {\rm{\Omega }}|}\,{\rm{for}}\,x\in \partial {\rm{\Omega }},$$

which is the compatibility condition, obtained by integrating Poisson’s equation (4) over Ω.

Debye’s^15^ concept of charge screening, which makes the induced field decay exponentially fast away from a charge, does not apply when electroneutrality is broken and long-range correlations lead to a gradient of charges, as is the case, for example, in a ball without inward directed current. By solving (8) numerically and asymptotically, a new capacitance law was derived for an electrolytic solution in a ball of radius *R* ^14^, where the difference of potentials between the center *C* and any point of the spherical surface *S*, *V*(*C*) − *V*(*S*), increases with the total number of charges, first linearly and then logarithmically

$$V(C)-V(S)\approx -2\frac{kT}{ze}\,\mathrm{log}\,\frac{2\pi R{(ze)}^{2}N}{\varepsilon {\varepsilon }_{0}kT|\partial {\rm{\Omega }}|},$$

The effect of the geometry on the voltage and the charge distribution for other cell shapes is described below.

### Local boundary curvature affects field and charge distribution

**Figure 1:**
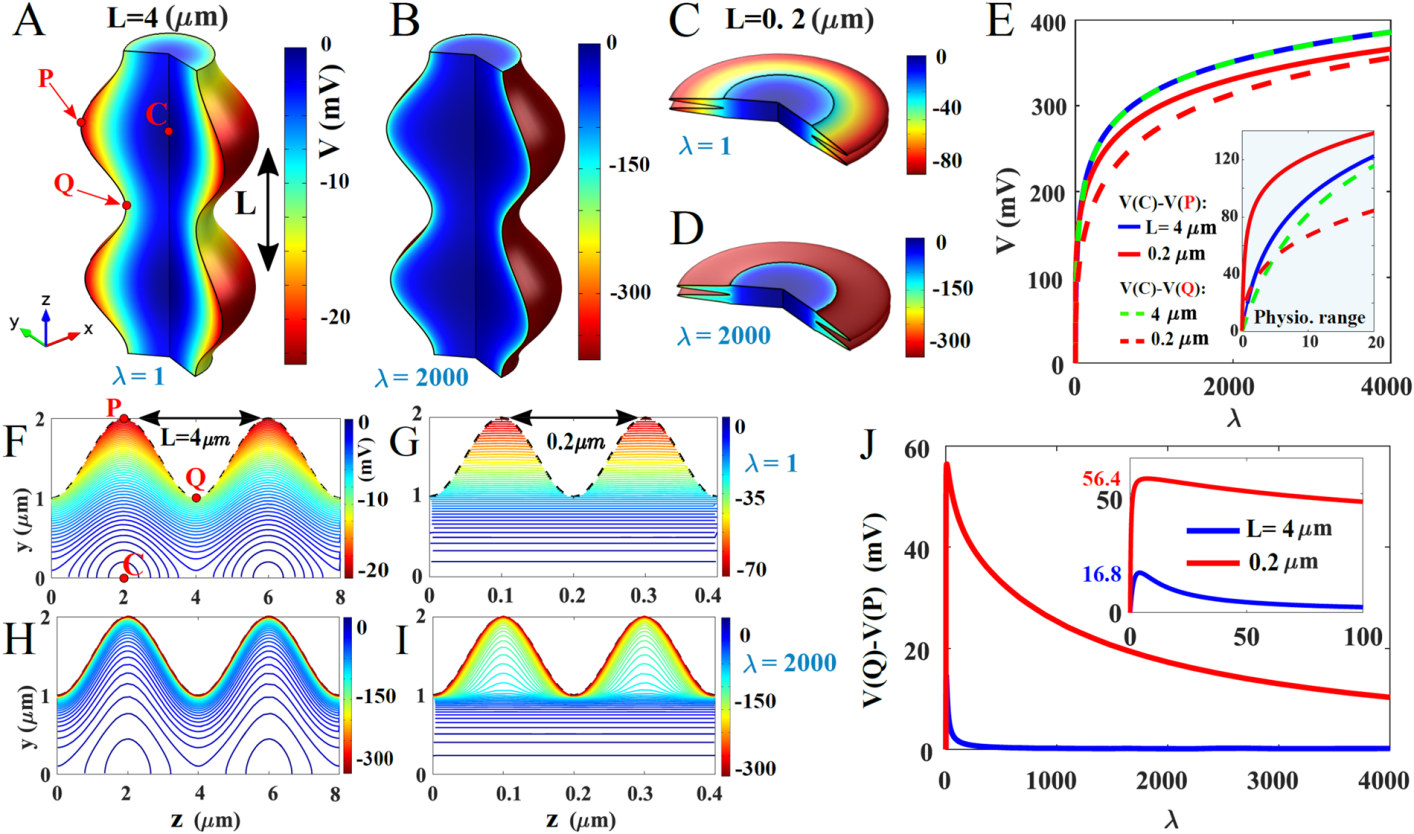
Numerical evaluation of voltage distribution in a corrugated cylinder (**A**–**D**). Voltage distribution computed for *λ* = 1 (**A**–**C**) and *λ* = 2000 (**B**–**D**). The boundary of the cylinder around the symmetry *z*− axis is defined by the curve *γ*(*z*) = 1 + 0.5 sin(2*πz*/*L*), where *L* is a parameter (*L* = 4 *μm* (**A,B**), and *L* = 0.2 *μm* (**C**,**D**). (**E**) Voltage differences *V*(*C*) − *V*(*Q*) (dashed line) and *V*(*P*) − *V*(*C*) (solid line) versus *λ*, computed for *L* = 0.2 *μm* (red) and *L* = 4 *μm* (blue, green), where *P* (resp. *Q*) is the maximum (resp. minimum) of the curve *γ*(*z*) and *C* is defined by *V*(*C*) = *min*(*V*).The inset panel in (**E**) represents a magnification of the range of *λ* corresponding to physiological values of the voltage. (**F**–**I**) Isopotential lines in the *yOz* plane, computed for various (*λ*, *L*): (**F**) (1, 4), (**G**) (1, 0.2), (**H**) (2000, 4), and (**I**) (2000, 0.2). (**J**) Voltage difference *V*(*Q*) − *V*(*P*) versus *λ*, computed for *L* = 4 *μm* (blue) and *L* = 0.2 *μm* (red). The inset in panel **J** is a magnification of the small *λ* region.

Axons and dendrites are not perfect cylinders and the curvature of their surfaces has many local maxima^16^. It turns out that this local curvature can influence the local voltage significantly, as shown in numerical solutions (using Comsol classical packages) of the PNP equations (Fig. 1), which reveal that regions of high curvature correspond to local charge accumulation. This effect should be sufficient to influence the voltage by creating measurable local voltage increase of the order of a few millivolts. The voltage inside the cylinder and along curved surfaces can vary (Fig. 1A–D) and can also depend on changes in curvature (Fig. 1F–I) and in the total number of charges *N* (Fig. 1E–J). Curvature creates a narrow boundary layer in the voltage (Fig. 1H,I).

**Figure 2:**
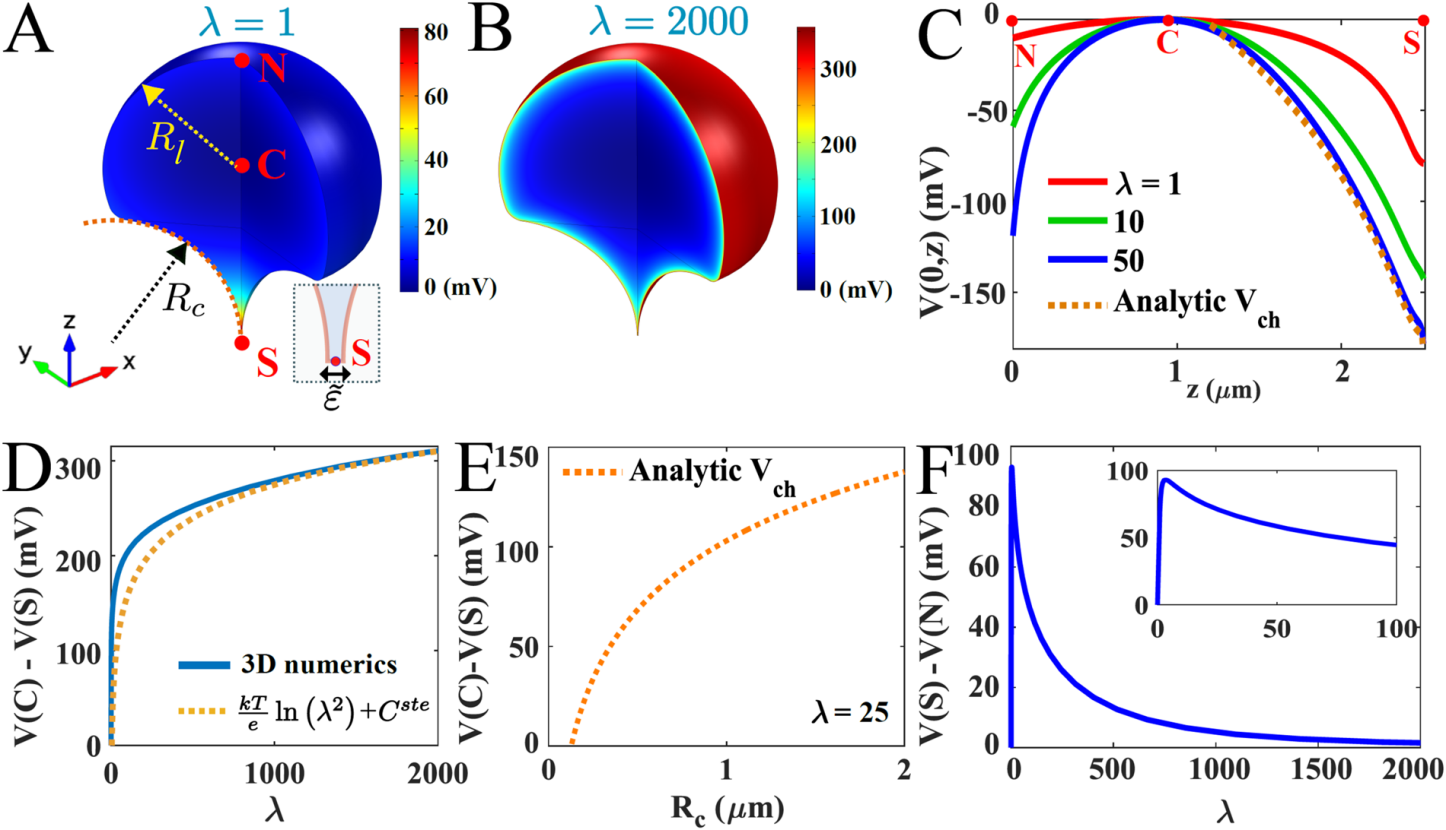
Distribution of charge and voltage in a domain with a narrow funnel. (**A**,**B**) Voltage distribution obtained for *λ* = 1 (**A**) and *λ* = 2000 (**B**), both for $\tilde{\varepsilon }=0.01\,\mu m$. (**C**) Voltage distribution evaluated along the *z*- axis, for *λ* = 1 (red), *λ* = 10 (green), *λ* = 50 (dashed blue) and obtained analytically (dotted orange) with formula (18). (**D**) Difference of voltage between *V*(*C*) − *V*(*S*) versus *λ* (blue) compared to the logarithmic function $\frac{kT}{e}ln{\lambda }^{2}+{C}^{ste}$, where *S*, *N* and *C* are the south, north pole and the center of mass respectively. (**E**) Difference of voltage between *V*(*C*) − *V*(*S*) versus the curvature radius *R*_*c*_ of the cusp-shape funnel, computed from eq. (18) for *ε* = 0.01 and *λ* = 25. (**F**) Voltage difference *V*(*N*) − *V*(*S*) between the two poles versus *λ*. The inset in panel (**E**) is a magnification in the small *λ* region.

The previous computations assume one specie only and in practice, due to the presence of *N* other ions (i.e., *λ* = (*ze*)^2^ *N*/*εε*_0_ *kT*), should only represent the excess of positive charges. Thus, we explore a large range for the parameter *λ* (a generalization of Bjerrum length *l*_*B*_ = (*ze*)^2^/*εε*_0_ *kT*, but has no physical units in dim 2 and is a length in dim 3). Note that *λ* is not simply given by the difference of positive and negative charges, due to the non-linear PNP equations. From the exploration of the graphs in Figs 1 and 2, we expect *λ* ≈ 1–500, which corresponds to a range *N* ≈ 10^2^–5 · 10^4^, leading to voltage fluctuations of few tens of mV. A more physiological range for the parameter *λ* is shown in Fig. 1E (inset). Consequently, the resting cross-membrane potential and the resting voltage across voltage-gated channels along a dendrite may vary, depending on curvature. This may affect the propagation and genesis of local depolarization or back-propagation action potential in dendrites of neuronal cells^17^.

### A cusp-shaped funnel can influence charge distribution

The passage of ions and other particles between different cellular compartments is done through narrow passages that form cusp-shaped funnels, which have negative curvature (see the example described in Fig. 2A). We consider here the case of dendritic spines, whose geometry is approximated as a convex domain (head) connected by a cylindrical neck to the dendritic shaft. This geometrical model approximation was already validated for diffusion^18^. We observe that the cusp-shaped funnel prevents the entrance of charges, at least when their number does not exceed a given threshold (Fig. 2C–F). Therefore, there is a difference of steady state potential drop between the end of the funnel and the bulk of the domain.

Assume that for a dimensionless domain ${\rm{\Omega }}\subset {{\mathbb{R}}}^{2}$ with a cusp-shaped funnel *F* formed by two bounding circles of equal radii *R*_*c*_ (see Fig. 2A), the opening of the funnel is $\tilde{\varepsilon }\ll 1$ (in unit of micrometers). This radius is used in changing to dimensionless coordinates, so we may assume *R*_*c*_ = 1. The surface area of the domain boundary is |∂Ω|. For an uncharged funnel domain, the condition (5) becomes ∂*V*(*x*)/∂*n* = 0 *for x* ∈ *F*. The potential drop between the center of Ω and the end of the cusp is found by solving (8) in a domain obtained by mapping Ω conformally by the Möbius transformation^18^

10 $$w=w(z)=\frac{z-\alpha }{1-\alpha z},$$

where $\alpha =-1-\sqrt{\tilde{\varepsilon }/{R}_{c}}+O(\varepsilon )$ and *R*_*c*_ is the (dimensional) radius of curvature at the cusp domain (Fig. 2A). In the variable *w* = *Re* ^*iθ*^ = *X* + *iY*, the dimensional solution in the image of the cusp is given by

11 $$\begin{array}{rcl}V(\theta ) & = & \mathrm{ln}\,\sqrt{\frac{{R}_{c}}{\tilde{\varepsilon }}}-\,\mathrm{ln}\,[\frac{1}{{\pi }^{2}}\,\mathrm{ln}\,\frac{{\pi }^{6}|\partial {\rm{\Omega }}|{R}_{c}^{\mathrm{1/2}}}{{2}^{3}{\tilde{\varepsilon }}^{\mathrm{3/2}}}]-\,\frac{2\theta }{{\pi }^{2}}\,\mathrm{ln}\,[\frac{{\pi }^{6}|\partial {\rm{\Omega }}|{R}_{c}^{\mathrm{1/2}}}{{2}^{3}{\tilde{\varepsilon }}^{\mathrm{3/2}}}]\\  &  & \,\times {\rm{arc}}\,\tan (\sqrt{\frac{{R}_{c}}{\tilde{\varepsilon }}}\theta )+\,\mathrm{ln}\,[{\cos }^{2}\frac{1-\frac{\mathrm{4|}\partial {\rm{\Omega }}|}{\lambda \pi \sqrt{\tilde{\varepsilon }{R}_{c}}}}{2}\theta ]\mathrm{.}\end{array}$$

Note that in the planar case *λ* has no dimensional units. It leads for $\lambda \gg 1$, to the dimensional voltage drop

12 $$V(S)-V(C)=\frac{kT}{ze}\,\mathrm{log}(\frac{{2}^{5}|\partial {\rm{\Omega }}|\sqrt{\tilde{\varepsilon }}}{{\pi }^{6}{\lambda }^{2}{R}_{c}^{\mathrm{3/2}}})+O\mathrm{(1).}$$

This difference should be compared with that between the north pole *N* and the center *C*, given in ref. 14, is given by

13 $$V(N)-V(C)=\frac{kT}{ze}\,\mathrm{log}\,{(\frac{8\pi }{8\pi +{\lambda }_{D}})}^{2}\mathrm{.}$$

where *λ*_*D*_ = 2*πλR*_*l*_/|∂Ω| (*R*_*l*_ is the (dimensional) radius of the external domain Ω). For a three-dimensional symmetric domain with a cusp-funnel,

14 $${\lambda }_{3d}=\lambda {R}_{C}\,{\rm{and}}$$

15 $${V}_{3d}(\theta )=V(\theta )+O(\sqrt{\tilde{\varepsilon }/{R}_{c}}\mathrm{).}$$

We conclude in the limit of $\lambda \gg 1$ and $\tilde{\varepsilon }\to 0$ that the difference of potential between the end of cusp *S* and the north pole *N* in the domain is obtained by adding (13) and (12) and we get

16 $$\begin{array}{rcl}V(S)-V(N) & = & \frac{kT}{ze}\,\mathrm{ln}\,(\frac{{2}^{5}|\partial {\rm{\Omega }}|\sqrt{\tilde{\varepsilon }}}{{\pi }^{6}{R}_{c}^{\mathrm{3/2}}})\\  &  & +\,2\frac{kT}{ze}\,\mathrm{ln}\,(\frac{{R}_{l}}{\mathrm{4|}\partial {\rm{\Omega }}|})+O(\frac{1}{\lambda })\mathrm{.}\end{array}$$

When Ω is charged, the boundary condition is still (9) and the potential drop is

17 $$V(S)-V(N)=2\frac{kT}{ze}\,\mathrm{ln}(\frac{\pi {c}^{2}{R}_{l}}{16\,{R}_{c}}),$$

The constant *c* depends only on the geometric center of mass *C* such as $w(C)=c\sqrt{\frac{\tilde{\varepsilon }}{{R}_{c}}}$, where *w* is the Möbius transformation (10), *R*_*c*_ is the curvature at the cusp and $\tilde{\varepsilon }$ the width at the base of the cusp. The analytical solution in the charged cusp-funnel domain is (in the same conformal coordinates as in eq. 11)

18 $${V}_{ch}(\theta )=\{\begin{array}{c}-\frac{kT}{ze}\,\mathrm{ln}\,\frac{|{e}^{i\theta }-{\mathrm{1|}}^{4}}{8}{(\frac{\lambda \pi {R}_{c}}{\mathrm{2|}\partial {\rm{\Omega }}\mathrm{|(1}-\cos \theta )+\lambda \tilde{\varepsilon }})}^{2},\,{\rm{for}}\,\,\theta \in [\mathrm{0,}\,\pi -\sqrt{\frac{\tilde{\varepsilon }}{{R}_{c}}}]\\ \frac{kT}{ze}(\mathrm{ln}\,{\cos }^{2}\frac{\pi \sqrt{{R}_{c}}}{2}\frac{|\theta -\pi +\sqrt{\frac{\tilde{\varepsilon }}{{R}_{c}}}|}{\sqrt{\tilde{\varepsilon }}}(1-\frac{\mathrm{2|}\partial {\rm{\Omega }}|}{\lambda \tilde{\varepsilon }})-2\,\mathrm{ln}\,\frac{\sqrt{2}{R}_{c}\lambda \pi }{\mathrm{4|}\partial {\rm{\Omega }}|+\lambda \tilde{\varepsilon }}\,,\,{\rm{for}}\,\theta \in [\pi -\sqrt{\frac{\tilde{\varepsilon }}{{R}_{c}}},\pi ])\end{array}$$

which is in very good agreement with the numerical solution (Fig. 2C dotted orange). The present results are quite different from the Gouy-Chapman theory which concerns the exponential decay of voltage away from the double layer on a flat surface and derived for electro-neutral bulk, which is not the case here (see wiki “double layer”). It follows that the funnel curvature is an interesting free parameter for the design of optimized nano-pipettes to regulate the molecular and ionic fluxes. These pipettes were recently designed to record voltage in dendritic spines^19^. Thus considering the shape of the pipette as a new parameter to control the ion fluxes injected in small biological domain, could impact the design and the precision of future biological experiments.

### Voltage distribution in an elongated ellipse

**Figure 3:**
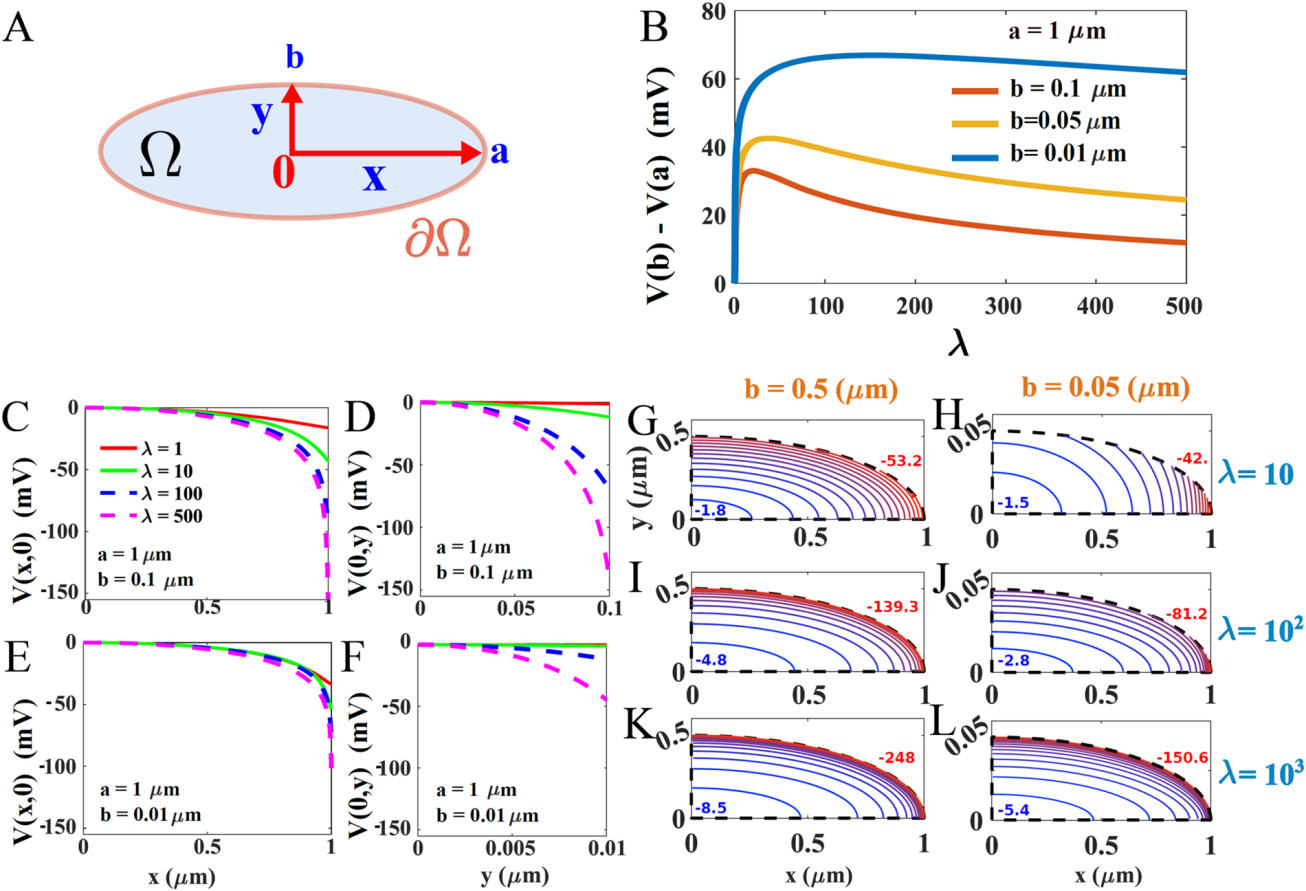
Voltage distribution computed numerically in a narrow ellipse (**A**). Representation of the elliptic two-dimensional domain Ω, where *a* and *b* are the major and the minor axis respectively. (**B**) Voltage difference *V*(*b*) − *V*(*a*) versus *λ*, obtained for *a* = 1 and *b* = 0.1 (red), *b* = 0.05 (yellow) and *b* = 0.01 (blue). (**C**,**E**) Voltage distribution computed along the *x*-axis for different values of *b* and *λ*: *λ* = 1 (red), *λ* = 10 (green), *λ* = 100 (dashed blue) and *λ* = 500 (dashed magenta). (**D**,**F**) Voltage distribution computed along the minor axis *Oy*, for *b* = 0.1 (panel D) and *b* = 0.01 (panel F). (**G**–**L**) Equipotential contours computed for *a* = 1 and varying (*b*, *λ*). The red values represent the minimal potential while the blue ones are the maximal potential.

Numerical solutions of the PNP equations 8 (Fig. 3A) show the effect of local charge accumulation in an elongated ellipse, in particular the potential difference *V*(*A*) − *V*(*B*) is maximal on the ellipse for *A* and *B* the ends of the small and long axes (Fig. 3B,C). The voltage along each axis is shown in Fig. 3D–G. The elliptic domain can represent the cross section of an axon, and confirms the effect of curvature discussed in the previous sections. Specifically, that charges accumulate near the boundary of highest curvature. The equipotential contours are shown in Fig. 3G–L.

## Discussion and Conclusions

As shown in the present study, inside a non-flat electrolyte domain characterized by significant changes in membrane curvature, the difference of voltage varies with the log of the curvature. Specifically, we derived various formula 16, 17 and 18 at a cusp-shaped funnel (curvature) *R*_*c*_ and we showed that the changes are significant in the range *R*_*c*_ ∈ [0.1–2] *μm* (Fig. 2E). These formula should be considered as predictions to study the effect of curvature inside neuronal microdomains, such as dendritic spines or part of dendrites.

We found here that local changes in geometry, non-electro-neutrality, and a dielectric boundary affect charge distribution in electro-diffusion of electrolytes, as previously suggested in refs 13, 14 and 20. This paper considers a single ionic specie, whereas in reality the solution contains multiple positive and negative ions. The present electro-diffusion model captures the effect of the excess of positive ions. Consequently, the large value of these voltage differences that we have found in the large charge limit *λ* → ∞ are probably much attenuated in a mixed positive and negative ionic solution, but the electro-neutrality remains broken. For large *λ*, the largest voltage differences occur near the boundary as shown in Figs 1B–D–I and 2B, and Fig. 3K, but this property will certainly remain true even if the difference between positive and negative ions decreases. The assumption of non-electroneutrality is based on the known cytoplasmic ionic concentrations given by *Na* ^+^  = 12 *mM*, *K* ^+^ = 155 *mM* and *Cl* ^−^ = 4.2 *mM* ^6^. There is a clear unbalance toward positive charges. Are negative charges missing? Probably there are molecules of various sizes with negative charges, but their diffusion coefficients are certainly much smaller than these of the ions. This difference of mobility is certainly a key feature in maintaining non-electro-neutrality and should be investigated both by ionic numerical simulations of electrolytes and experimentally. To conclude, the present analysis by considering only one positive specie, leads to an excessive potential difference, that will be diminished when adding negative charged proteins, but it provides a new qualitative insight, suggesting that the geometry of dendritic spines can influence significantly the voltage amplitude and shape of the excitatory-post-synaptic-potential, which depends on the membrane curvature. This prediction suggests that the neuronal code and communication is directly modulated by the geometry of nano-micro synaptic domains. This effect could have consequences in other electrical cellular domains, such as cardiac myocytes, glial protrusions, cilia and more. Indeed, local curvature is associated with a gradient of charge density that can affect the electrical properties of micro-compartments^10, 11, 21–24^. In all cases, charge accumulates near boundary points of locally maximal curvature.

These results and predictions can further be used to design nano-devices such as pipettes and to better understand voltage changes inside dendrites and axons. Finally, the non-electroneutrality could also be amplified during a synaptic current flow with an amplitude of 50 pA during 5 ms, leading to an entry of roughly 1.510^6^ (positive charges), which cannot be compensated with the membrane capacitance. Indeed, the capacitance effect could at most neutralize 1.6% of the positive charges (the value is computed from the formula ${N}_{m}=\frac{{\varepsilon }_{0}{\varepsilon }_{r}{S}_{urf}}{de}V$, with a dielectric membrane thickness d = 8 nm, the electric permittivity *ε*_0_ *ε*_*r*_ = 4.410^−11^ *F*/*m*, *ε*_*r*_ = 5, the membrane potential V = 60 mV and the surface ${S}_{urf}\approx 4\pi {R}_{bulk}^{2}$). Future analysis should reveal the charge distribution generated by a transient current.
